# Health Tract, No. 117

**Published:** 1864-11

**Authors:** 


HEALTH TRACT, No. 117.

### THE SOLDIER'S ALL.

It was a cheerless autumn day; the rain was falling in torrents; every thing was saturated with water; and as my wife passed among the sick and wounded and dying and dead soldiers, she bent over the wretched pallet of one, and asked him if he needed any thing. "Nothing, Madam, I thank you."

"Do you want any thing to read—any books, or papers, or magazines?"

Reaching his poor, sunburnt, scrawny hand from under the bed-clothing, he laid it on a book, and directing her attention to it, said: "This is all the reading I want."

It was a well-worn Bible. Happy man! A stranger, far from home, sick, in rags, apparently "not far" from the grave, he had no wants which his Bible could not supply. There were dark clouds in the sky above; his Bible was sunshine to him.

He knew nobody; nobody knew him; he was literally "a pilgrim and a stranger;" but he had an acquaintance in his Bible, and as he read it, his eyes fell upon old familiar names, which carried his mind back to the village-church, to the "family worship" of his childhood, and he read of David and of Jonathan, of Moses and of Elias, of Peter and of Paul, but most of all, of Jesus of Nazareth, the Friend of sinners and the Saviour of man.

Weak and wan as he was, he asked for no wine to sustain him, no delicacies, prepared by tender hands, to nourish him into life again; for he had "meat to eat" which those around him "knew not of." He read in his Bible morning, noon, and night; and he found out that as often as he read it, he felt nourished and comforted. It was a dish of which he never became tired; for although apparently the same, he found something new in it every day; some sweetness that he had not tasted before. No wonder, then, that he found every want supplied in the soiled book which he carefully kept always in reach.

Some soldiers, in tents and under other forms of shelter, were writing letters, turning over the leaves of magazines, or reading newspapers; but this soldier's Bible supplied all the reading he wanted. In it he found "things both new and old;" he found them reliable; to-day brought no contradiction of what he read yesterday. The messages which he received were telegraphed from heaven, and he had heard the "Operator" there say over and over again, as to his messages: "If it were not so, I would have told you." Happy soldier! Blessed book! Doubtless he would feel a full accord with him who wrote:

"This little book I'd rather own
  Than all the gold and gems
That e'er in monarch's coffers shone,
  Or all their diadems.
Yes! were the seas one chrysolite,
  The earth one golden ball,
And diamonds too the stars of night,
  This book were worth them all."

Reader, you will be sick one day; it may be a long sickness; you may be far from home, far from friends, far from medical aid. Let me tell you there is a "balm" in the Bible; a medicament, a cordial, of a nature so searching, of a power so all-pervading, that there is "no sorrow which it can not heal," no suffering which it can not soothe, no pain which it can not mitigate. It helped the soldier to live above his sufferings; it will help you to do the same. It met all his wants; it will meet yours. There was a fullness in it to him; that fullness it will be to you, and in an hour, too, when no human drug can avail, when the most skillful physician in the land is powerless to ease a single agony, and when the most that the best friends on earth can do for you, is to gaze at your suffering in sorrowing helplessness. Make the Bible then your companion, your counselor; keep it always in easy and convenient reach, as the soldier did; and learn like him to be satisfied in its fullness, and like him, to find in it a safe Guide, a Friend in need, and an able Physician.

